# Navigating language discordance in public health care in rural South Africa: a qualitative descriptive study of occupational therapists’ perspectives

**DOI:** 10.1186/s12913-023-09658-3

**Published:** 2023-08-15

**Authors:** M. C. Ramafikeng, E. Marshall

**Affiliations:** 1 School of Health and Social Care, University of Essex, Wivenhoe Park, Colchester, CO4 3SQ UK; 2 Marshall Occupational Therapy, 3rd Avenue, Kenilworth, 7708 Cape Town, South Africa

**Keywords:** Language discordance, Occupational therapy, Communication strategies, Quality health services

## Abstract

**Background:**

Language discordance occurs in healthcare when staff and service users do not share proficiency in the same language. It is a global phenomenon impacting on the quality of health services, as person-centred practice requires communication to establish partnerships and rapport. In a country as linguistically diverse as South Africa, effective ways to navigate language discordance in health care are urgently required, yet there is limited research. This study aimed to describe how occupational therapists navigated language discordance when working in the public health sector in KwaZulu-Natal.

**Methods:**

A qualitative descriptive design involved using purposive sampling to recruit occupational therapists as participants (*n* = 8) for 15 semi-structured interviews. Thematic analysis was used to analyse the data, which included reflective journal entries from all participants.

**Results:**

The four emergent themes were: (1) concurrent use of strategies, (2) *I’m doing as much I can, what more can I do?* (3) *Language definitely impacts that therapy process* and lastly, (4) systemic oppression perpetuating language discordance.

**Conclusion:**

Language discordance is a complex context-specific phenomenon, therefore insight into concurrent use of strategies is important to practitioners to enable them to navigate language discordance and ensure provision of quality services. These insights are significant for healthcare professionals and resource allocators as they shed light on the shortcomings of advocating for a single strategy such as providing trained interpreters. Successful navigation is characterised by determination, being kind to oneself, willingness to learn and use of pragmatic and flexible approaches. To prepare to navigate language discordance in a low-resource context, education should extend in time and scope, to include multiple strategies, culture and relevant languages.

## Background

Language discordance is a global phenomenon [1] in which health professionals and service users do not share proficiency in the same language [2]. The emphasis on proficiency contrasts with the notion of a language barrier, which often implies an insurmountable problem with the service user. Weng and Landes [3] have extended the definition of language discordance to include other means of communication such as body language and braille, pointing out how these are also shared in communities. The incidence of language discordance is likely to be on the rise as more people, including health professionals, move from their home countries or regions to other places where their home languages are not widely spoken. For example, migration has increased the prevalence of language discordance in Europe [4], North America [5], the Middle East [6], and Asia [7, 8]. In the South African context, immigration, internal migration, and largely, linguistic heterogeneity of the society, account for experiences of language discordance.

Language discordance has been reported to result in; difficulties in gaining informed consent [8, 9], incomplete assessments [10, 11], diminished rapport building [12, 13], and safety risks [8, 14]. Studies from South Africa show that poor communication between health professionals and service users stunted the appropriateness and acceptability of health care [15], especially in rural areas [16]. Language discordance has a direct impact on ‘process’ and subsequently the ‘outcome’ of quality of care as outlined by Donabedian [17]. Quality of care has three components; 1) *processes,* referring to the care that the health professionals provide, 2) *structure*, which includes staffing, the institutional culture, policies, and the physical environment, and 3) *outcomes*, being the result of the care processes, provided within the specific structure [17]. The quality of health care is dependent on several factors such as efficiency, effectiveness, person-centredness, safety, and sustainability [18]. These factors resonate with Donabedian’s [17] description.

Although language discordance is experienced by all health professionals, the phenomenon is under-explored within occupational therapy literature, particularly in South Africa where there is significant linguistic diversity that contributes to inequalities in health services received and rendered. The country is described as a melting pot of ethnicities, music, cuisines, landscapes, and languages [19]. The National Language Policy Framework [20] states that there are about twenty-five languages spoken in South Africa, with eleven gaining official status in 1996. About 78% of the population speak an indigenous South African language as their home language [21]. However, English and Afrikaans, which are historically associated with colonialism and oppression, continue to be privileged in post-Apartheid sectors [22, 23], such as the healthcare sector [24]. Exploring language discordance is relevant for occupational therapy as services are founded on partnership, trust, and rapport, which all require communication to be established. Therefore, language discordance could impact the client-centred nature and quality of the services rendered.

Several strategies used to mitigate the effects of language discordance have been reported. These include use of formal (telephonic included) [11, 12] and informal interpreters [8, 11, 25], staff attending short-term language courses [26], professionals communicating using basic language and non-verbals [3, 27], hiring more linguistically diverse staff [3, 11, 12], and translation [28]. However, these strategies have varying levels of effectiveness, ethical implications, and different logistical requirements across both well-resourced and under-resourced contexts. There is also limited evidence on the concurrent use of the strategies outlined above.

Professional reasoning and creativity are important in guiding decisions on selection of strategies to use concurrently as well as when to employ which combination of strategies. This decision making is important in a rural South African context, where resources for navigating language discordance may be limited, adversely affecting the strategies used by health professionals. While there are insights in the literature, occupational therapists are rarely specifically mentioned. This paper therefore reports on a study whose aim was to describe strategies that occupational therapists working in the public health sector in KwaZulu-Natal use to navigate language discordance [29]. This study was the first study exploring strategies used by occupational therapists to navigate language discordance, therefore bridging a gap in literature. The aim of the paper is to report on the experiences of navigating language discordance during occupational therapy service delivery.

## Methods

### Study design

A descriptive qualitative design [30] was used to create a detailed and holistic account of the topic [31], in contrast to interpretation or building theory in exploratory research [32]. As a process, navigating language discordance involves reflection and reasoning which are not easily observed. Hence the design involved collecting qualitative data from participants, via semi-structured interviews [30]. Two interviews per participant were conducted, to give more scope for capturing details and to ensure that saturation is reached. Thematic analysis of the data enabled parallels and contrasts between individual experiences to be drawn, synthesising an account of navigating language discordance and the implications for services. The researcher approached this study with the assumption that reality is subjective, socially constructed and there are multiple perspectives to language discordance as the phenomenon of interest in this study. These assumptions reflect a constructivist paradigm [33] and a relativist ontology [34], hence a naturalist qualitative methodology was adopted in this study as evident in the design, data collection and analysis methods used.

### The researcher

The second author (EM) conducted the research towards her post-registration Masters degree [29]. She was under the supervision of the first author (MR). As a white, English speaking, middle class, female occupational therapist, the second author (EM) had personally experienced language discordance in a similar setting and realised how crucial comprehensive communication was for quality occupational therapy care and her pursuit for greater access to quality care motivated her to invest in this topic. The researcher did not know the participants prior to this study.

### Study setting

This study was conducted in one of the ten districts of the Kwa-Zulu Natal province in South Africa [35]. In this setting, occupational therapists were more likely to be navigating language discordance for several socioeconomic reasons. About 90% of the population speak isiZulu as their home language [36] with only about 32% having matriculated or obtained higher qualifications [37]. The district is largely rural, with just under 70% of the population being employed, largely as farm workers and 63% of the population live below the food poverty line [35].

Although 73% of the South African population [38] access the public health sector, only 54% of the health budget is allocated to it [39]. As a result, there is understaffing and a lack of resources. Public health care encompasses three tiers: (1) primary health care clinics, (2) district hospitals and (3) larger tertiary specialist hospitals. These free services are available to all citizens with no formal health insurance plans or the ability to pay for private health care [40]. Most primary health care clinics are the first point of contact, staffed by nurses providing community services within 5 km and district hospitals offer more investigations, treatment and minor procedures [41]. All South African graduates in the health professions are required to complete community service to be licensed to practice [42]. Community service, which is usually a year in district general hospitals in rural and underserved areas, brings language discordance into sharp focus. Most of the participants were based at district hospitals, with some providing outreach to the primary health care clinics.

Ethical approval was obtained from the Human Research Ethics Committee of the Faculty of Health Sciences at the University of Cape Town (HREC 081/2020) and from the KwaZulu-Natal Department of Health (KZ_202002_023) prior to the recruitment process. The research was conducted according to the Declaration of Helsinki [43] guidelines for conducting research with human subjects.

### Recruitment process

The district occupational therapy representatives acted as gatekeepers, through which the potential participants could be contacted. Invitations to partake in the research were emailed to 25 occupational therapists that worked in the public health sector in the district. In total, 11 occupational therapists expressed interest, but one did not meet the inclusion criteria. The criteria were 1) being an occupational therapist with 2) experiences of working in the selected district in the last five years (2015–2020) and 3) language discordance. Purposive sampling [44] the homogenous type, was used to recruit a target sample size of eight participants and recruitment stopped when the target was reached. Based on the plan for conducting two interviews per participant, therefore generating 16 interviews, the sample size of eight was considered sufficient for reaching data saturation. A systematic review on sample size and saturation [45] supports Guest et al. (2006)’s [46] observation that data saturation usually occurs with twelve interviews. Homogenous sampling is used when the emphasis is on the similarity in characteristics of the sample, therefore reducing variation [47] such as was the case for this study. The focus was on shared characteristics of being an occupational therapist who worked in the district and had experienced language discordance. The information sheet and consent form were emailed to the participants, and they returned the signed consent form to the researcher before their first interview. They were also given an opportunity to ask questions about the study via email and Whatsapp, which the researcher responded to openly. There were no dropouts in this study.

### Participants

The eight participants were all female, aged 23–35 (in 2020) and had one to seven years of experience in the public health sector. Six of the participants spoke English as their home language apart from two participants as captured in Table 1 below. The majority (*n* = 6) of the participants spoke Afrikaans as their second language. The participants identified their own pseudonyms which are used throughout this paper.

**Table 1:** Demographic characteristics of participants

**Pseudonym**	**Work experience in public health sector (Years)**	**Age (Years)**	**Population group**	**Home language**	**Second language**
Libby	7	35	White	English	Afrikaans
TDubbs	1	26	White	English	Afrikaans
Riley	1	26	White	English	Afrikaans
Cassandra	1	23	Coloured	English	Afrikaans
Demi	1	27	Coloured	English	Afrikaans
Monica	2	25	African	isiZulu	English
Harsha	1	25	Indian	English	Afrikaans
Moh	1	28	African	Sesotho	English

### Data collection

**Fig. 1:**
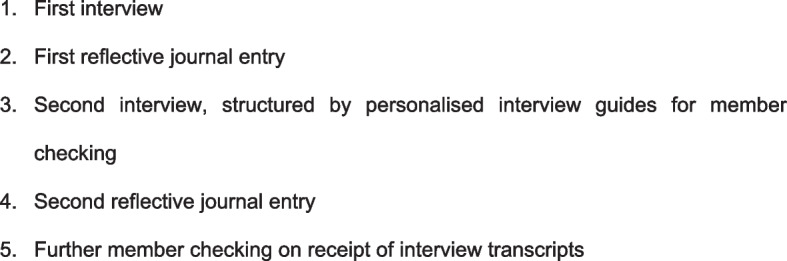
Details of procedure for participants

The procedure for data collection is indicated in Fig. 1. Due to the COVID-19 pandemic data were collected virtually from May to July 2020. Originally it was planned to host focus groups for reflective discussions to supplement the interviews. Reflective journals were a viable alternative due to the social restrictions. Reflective journaling is a tool that aims to foster reflection and introspection [48] and is an effective way to obtain information about a person’s feelings [49]. The participants could add more experiences and capture their reflections after the interviews, prompted by the researcher, who used her field notes to create reflection points to send to each of them. Reflections were recorded digitally in a format of their choice (journal style, bullet points, voice note), and in as little or as much detail as they desired. The data from the journals were used to triangulate data, thereby ensuring credibility of findings [50].

In total, 15 individual interviews were conducted by the researcher alone, with each ranging from 45–75 min in duration. All interviews were conducted in English and were audio recorded. Each participant was interviewed twice, except for one participant who had the least experience of language discordance as she spoke the local language. After the first interview for this participant, the researcher felt that all questions had been answered, therefore a second interview was not required for this participant.

**Fig. 2:**
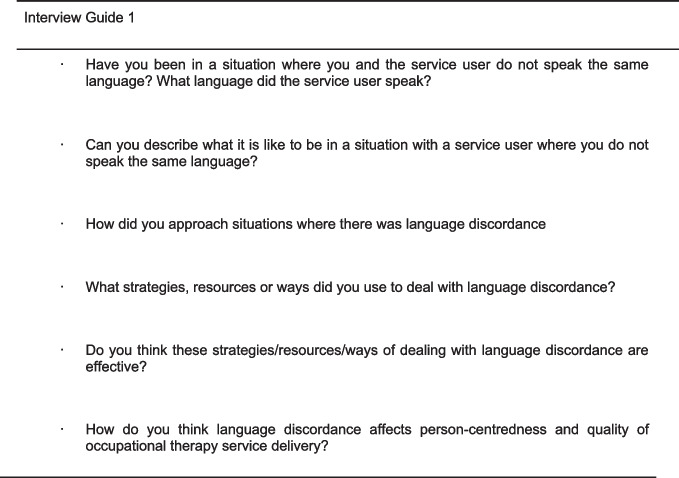
Guiding questions for first interview

Interview guides were used to structure the interviews, with the same one being used in the first interview with each participant (Fig. 2). For the second interview, the researcher tailored a draft guide for each participant, drawing on their first interviews and reflective journal entries. This approach facilitated data saturation and member checking. Data saturation was evident when repetition emerged in sharing by participants within and across the 15 interviews. Guest and colleagues [46] notes that data saturation usually occurs within the first 12 interviews and in this study, 15 interviews were done. As the participants also read through their interview transcripts and gave feedback, the two stages of member checking ensured credibility of findings [50]. Participants confirmed the accuracy of the transcripts and did not request changes or make further additions following the final member checking stage.

All interview recordings were transcribed by the first author and then reviewed by the second author, to ensure data immersion [50]. All data were stored securely in accordance with university policies.

### Data analysis

Interview data were analysed using thematic analysis, guided by Braun and Clarke’s [51] six-step process 1) become familiar with the data, 2) generate initial codes, 3) search for themes, 4) review themes, 5) define themes, and 6) write up. Thematic analysis enabled a detailed and nuanced description of the participants' experiences [51], in preference to content analysis which primarily describes a topic using concepts repeated in the data [52]. A line-by-line coding technique [53] was used for the step of generating codes resulting in 1461 initial codes being generated deductively using the research objectives as a guide [29]. The codes were consolidated into 11 sub-themes through an inductive process, and these were further collapsed into four main themes. The researcher’s field notes and reflections were used to cross reference findings during analysis, in a reflexive approach for credibility.

There was a consistent approach to ensuring quality. For credibility, in addition to the strategies already mentioned, peer briefing [50] was employed. Confirmability and dependability were ensured using an audit trail [54] that included having a clear research plan outlined within the research proposal that was submitted for ethical approval. This plan was followed throughout the research process, and direct quotes from the interviews and the reflective journals were included to support findings. Lastly, for transferability, a thick description of the research site [55] was provided.

## Results

The participants experienced language discordance as challenging, with linguistic and cultural components. Navigating the challenges required resilience. Four themes were generated from the data analysis, starting with the strategies used concurrently in practice. These strategies had limitations, but the second theme suggested participants were doing as much as they could to compensate. The third theme covered the impact on services. The participants reflected in the interviews and their journals on the factors perpetuating language discordance, forming the final theme.

### Concurrent use of strategies

The participants described using an interpreter as their usual strategy, often concurrently with attempting to use service users’ languages and other modes of communication. Participants did not have access to formal trained interpreters, therefore they worked with informal interpreters who were co-workers, family members, volunteers and in some cases other service users. Despite service users mostly speaking IsiZulu as their home language, English was used by co-workers as the bridging language and the dominant language of practice:

... *everybody there spoke English and understood English…so communicating with rehab or any other department wasn’t that difficult*” [Cassandra].

Co-workers were health professionals and support staff, such as security guards, cleaners, porters, and clerks, chosen depending on who was available at the time, their proficiency in English and their level of education.

Participants shared mixed views about interpreters, due to challenges with the quality of interpretation and professional conduct, especially when co-workers were not health professionals. Monica explained: *“I would ask a colleague or…another health professional, to help*. *It was also a lot easier”*. Some participants audio recorded answers from service users and obtained interpretation outside the therapy space. With co-workers who were not professionals, inappropriate conduct was a risk, as Demi commented:

I have to make sure that the translators wouldn’t laugh…or you know, they would have to be sensitive…

Conversely, when some physiotherapists acted as informal interpreters, there was a risk of losing control of the therapy process.

Nursing staff were often unwilling to help, as Riley indicated: “when you’re by yourself in a ward…90% of the time the nurses are not going to help you”. Moh, who was of African descent, experienced more resistance with some nurses asking: “how did you expect to survive in Kwa-Zulu Natal if you’re not gonna learn the language?” signalling intolerance of her limited isiZulu proficiency compared to practitioners of other race groups.

As interpreting was not part of their co-workers’ job descriptions, participants gave priority to seeking help with assessments or to confirm home programmes. Using co-workers in the same team was easier, whereas some participants had to be organised in advance to make sure co-workers were available.

Most participants thought it was advantageous for a family member to interpret, as they were often involved in sessions anyway. Riley suggested that when working with children “*as long as the parents understood me, they’d explain it to their own child in Zulu*”. Family members acting as interpreters could enhance collaboration between service users, other co-workers, other family members and community members which was valuable for holistic, quality care.

To communicate without an interpreter, translanguaging and demonstrating were used as concurrent strategies. Translanguaging involved participants speaking single words or short phrases in broken isiZulu or a combination of many South African languages. Libby described how: “*you kind of potter along in a bit of English, a bit of sign language, a bit of Afrikaans, a bit of acting and a bit of Zulu”.*

Despite having studied isiZulu at university, participants felt very unprepared so began diligently learning isiZulu:

*I wanted to be independent…and not have to rely on someone the whole time. That was mainly what pushed me into learning it* [isiZulu] *myself* [Harsha].

As well as being immersed in a Zulu-speaking work and home environment, participants listened to isiZulu Gospel songs, joined group learning sessions and developed their own dictionaries. Learning isiZulu was empowering and necessary, as a few words assisted in building rapport and conveying meaning.

Gestures, pointing, touch and reading body language were also used as participants relied heavily on non-verbal communication, such as demonstration: “I was using my painful Zulu…while demonstrating…, so it was actually two-fold, because obviously my Zulu wasn’t really enough” [Riley]. Service users’ comprehension could be assessed in their responses to demonstration, but for some people with psychiatric or cognitive symptoms, this strategy was less effective. However, the hands-on nature of occupational therapy could help: “*I just had to rely on my…skills and … make sure that the…the activity was so engaging that…speech wasn’t really…a thing*” [Demi].

Other alternatives included visual aids such as charts and pictures from assessment forms and home exercise programme materials. Online resources including videos were used although the accuracy was variable. Clinical files and reports provided useful information. TDubbs described how every effort would be made to navigate language discordance: “if one way doesn’t work you try another. You don’t just give up because you can’t speak, you can’t verbalise…there’s ways around it”.

### “I’m doing everything that I can, what more can I do?”

The second theme revealed that it was important for participants to recognise their own efforts which involved time, being kind to oneself, responsibility, and determination. Extra time was required at work and after hours to learn the service users’ language, including key phrases required for therapy. Finding and negotiating with an interpreter took extra time. Sessions requiring interpretation took longer as participants had to establish understanding for the therapy to be effective: “*maybe giving them that little bit of extra time to make it as good as another session would be”* [Harsha].

However, the shared feeling was that, while going the extra mile was time consuming and not always possible, it was part of navigating language discordance:

…trying to remember, and remind myself that I’m doing the best I can, with the…vocabulary that I have, [and] not put any unnecessary pressure on myself [Moh].

Being kind to oneself supported efforts to persist with learning. Attempts at speaking the service users’ language were appreciated, but sometimes frustration was palpable and regarded as reasonable: “I can understand why they can be frustrated too, and I should be very sensitive to that, knowing what they’ve gone through, even before they’ve walked through the door” [Riley].

Participants felt that they had a responsibility to speak as much isiZulu as possible to provide occupational therapy despite language discordance. Demi described that although she was not prepared initially, her problem-solving skills and flexibility developed through trial and error. Monica suggested undergraduate education prepared her to take responsibility for addressing the challenges:

During practicals in varsity, there would be a lot of things that would go wrong during a session. … the OT training I received equipped me with skills to adapt and problem solve [Reflective Journal I Entry].

As a South African, Libby described the culture of “’n boer maak ‘n plan” (a farmer makes a plan), making things work irrespective of obstacles. Determination to navigate language discordance and provide the best service combined being kind to oneself with exploring, learning, failing and trying again. Harsha explained: “you just tell yourself that you’re learning, all the time. …I’m going to learn from this and do better”.

### “Language definitely impacts that therapy process”

The participants’ determination persisted because the impact on the occupational therapy process was clear to them. This third theme covered the necessity of language, quality of care and demographic factors that influenced communication.

Although activity is the medium for occupational therapy, language facilitates interaction between the therapist and service user, starting with building rapport. As a first language Zulu-speaker, Monica described the impact of being fluent:

*Immediately when I’m greeting you…you are greeting me … . I can ask you what’s wrong or what can I assist you with …it’s easy for me to understand what is wrong with you, even before me assessing you…or before treatment*.

In contrast, Harsha described:

*the most difficult [thing] was building a rapport with my patient, who couldn’t speak the same language as me*.

In her reflections, Cassandra shared her difficulties in early interactions which made her feel inadequate and frustrated knowing her services could have been better. Libby also described the negative impact on quality:

*I know that if I could communicate in my home language that person would get a* ***much*** *better service than what I am currently offering* [Libby].

History-taking and the range of potential assessments were limited:

*Firstly your assessments are…simplified, your…treatment methods are limited, your treat-, your client factors are, are shortened … because how’re you gonna dive into all these things?* [Riley].

Libby had two examples of how the limited range of interventions had an impact on the service user experience of occupational therapy:

*Someone’s had their… hand half chopped off at work, you need to be able to coach them through the psychological process too, and then you can’t do that*.

*trauma processing, psychiatry, is kind of lost… you just don’t get that richness, …. The more limited your language, the more concrete your therapy is* [Libby].

It was also difficult to adopt a person-centred approach, as Cassandra indicated:

*I would say that language is an enabler... meeting the needs of the person … if you don't have that it just takes away from the quality of your service*.

If therapy was less person-centred, goal setting was more difficult:

*you’re going in your own direction, you’re having your own goals… instead of doing it together, and collaboratively* [TDubbs].

Language discordance made participants feel inadequate about their skills and the therapy they provided. Covering the bare minimum also potentially breached ethical principles.

Ethical dilemmas were reported by all participants, such as choosing between the benefits of having an informal interpreter present, against risking confidentiality:

It wouldn’t be right, for me…to exclude them [an interpreter] just because I want it to be private…informed consent includes providing information to a client in a language that they understand [Demi: Reflective Journal I Entry].

Participants recognised that the benefit gained from therapy was compromised by language discordance:

*I didn’t feel comfortable with…how the treatment went…or how the session went …because I knew that I could have done more…I could have gotten more out of them….* [Monica]

Another ethical concern, autonomy, was diminished by language discordance when goal setting:

*for us to help a patient achieve independence or achieve their goals...we need to know what the person wants...if you are not able…to ask the right questions... you're not able to meet... what they aim to do* [Cassandra].

There were varying perspectives on harm, with some participants being adamant that language discordance could be harmful due to reduced comprehension of home programmes, or care in general. Libby stressed that any harm was not intentional:

*And even when you can’t speak the language…I think intent goes a long way*.

Another dilemma was having children as interpreters when working with adults. Participants agreed that it was inappropriate to have children interpret sensitive information for their parents. Linking with the second theme, participants confirmed that they were doing their best while reasoning their way through the dilemmas.

Demographic factors also had an impact, with characteristics such as age, diagnosis and background, influencing communication. It was easier to communicate with children who were learning English at school or young adults who spoke English at work. Usually, English was used to bridge differences in home languages. However, older people were not often proficient in English and also spoke a purer, less colloquial version of isiZulu.

With preschool children, Libby found her proficiency in their home language was sufficient, especially as they could also connect through play, the medium for therapy. Whereas Cassandra was concerned that she lacked spontaneity and the ability to give praise which she thought was vital for paediatric occupational therapy.

Communication varied according to diagnosis. Simple physical diagnoses were associated with more straightforward assessment and treatment, so participants could rely more on demonstration and observation. However, verbal communication could be harder with service users with psychiatric or intellectual conditions could be harder:

*It's really difficult for people who don't understand what you are saying to understand what you want them to do* [Cassandra].

Monica added that greater fluency is required in certain situations, for example with “A*ny complicated hand injuries that have like, any nerve injuries or…tendon injuries.”* Despite being a first-language Zulu-speaker, Monica indicated that communicating in her home language could be challenging because health and occupational therapy-related terms could not be translated, even when speaking the same language.

Participants worked in a variety of rural and peri-urban locations, with varying attitudes towards language use. Libby found that service users in rural contexts were more willing to make do with broken languages, whereas she experienced resistance in the peri-urban context. However, Harsha observed:

*I think they understood, like, okay, it’s not your first language…and…we can see that you are trying to speak our language*.

Moh was even praised for speaking isiZulu very well despite it being her third language. In contrast, instances of frustration and outrage from service users were reported:

*I had a guy try and punch me* [laughs] *because I couldn’t speak Zulu. Uhm, so that sense of complete outrage of you can’t speak well enough for my liking so I’m going to hit you.* [Libby]

For service users from other countries such as Somalia and Mozambique, it was harder to find an interpreter., To minimise the impact, other strategies were used:

*Being able to be hands-on … we’re going to have to just revert to gestures and hand-over-hand…and getting a hug by the end of that from the parent* [Libby].

### Systemic oppression perpetuates language discordance

In South Africa, language was used to oppress and racially divide people. This theme outlines how systemic oppression perpetuates language discordance through factors attitudes towards learning languages other than English, cultural and linguistic nuances, and poor educational preparation.

Participants had observed resistance to learning other languages:

*Some people complained that we had to do it* [the isiZulu course] *for one term, but I just thought … they weren’t thinking about the context* [Riley].

Similarly white occupational therapists, despite working in Zulu-speaking contexts, chose not to learn isiZulu:

*It surprises me, the amount … working, English-speaking people, have been in government for fifteen years and they can barely speak Zulu…and it comes off as …arrogance* [Riley].

On reflection, Riley stated:

English first language speakers are not commonly known to put effort into learning other languages. I don’t know why? Probably a form of arrogance, laziness or the effects of systemic white supremacy [Reflective Journal I Entry].

Demi expressed that despite wanting to communicate with service users, she found isiZulu too hard to learn. When informal interpreters were readily available, there was no pressure to learn the language.

The participants recognised that language and culture are intertwined, important for understanding the embodied or proverbial meaning of words, saying;

*when …some of the Zulu women say…the world feels …I’m sore everywhere…and it’s [like] the weight of the world is on my shoulders* [Libby].

Moh suggested that fluency does not guarantee that understanding of contextual meanings. Similarly, TDubbs reflected on awareness of accent:

When saying some English words (i.e., porridge) without an isiZulu accent, a lot of the time, it was not understood. However, if I added an accent (i.e., ‘porrreeg’) generally patients had a better understanding [TDubbs: Reflective Journal II Entry].

Four of the non-Zulu- speaking participants grew up in KwaZulu-Natal, but their school either did not offer isiZulu as a subject or teaching from Grade 3 to Grade 9 was inadequate:

*Living in KZN, I was doing Afrikaans at school…and I live in KZN. …it’s not common for people to speak Afrikaans. Obviously Zulu is a more common language, yet I did so much time at school learning a different language.* [Harsha]

Some participants were not exposed to isiZulu (or isiXhosa) until their first year of university but felt that it would have been better closer to completion of their degrees. In addition, one module was not sufficient to learn a new language and the content was often irrelevant for practice:

*Nurses, physios, dieticians, radiographers…we were all, together, like learning Zulu together, …which is fine for the basics, but…not to learn, …for your job* [TDubbs].

Riley added:

*We had a Zulu course…with BComm students and … it was like an extension of conversational…because obviously they’re not going to go into all medical stuff when you’ve got BComm students sitting there*.

Cassandra and Demi explained that they were taught to rehearse isiXhosa and isiZulu scripts, but not how to make sense of responses. Poor educational preparation also perpetuated language discordance, because participants did not feel prepared for the context: *adapt, adapt, adapt if things aren’t working adapt…but you’re very rarely told how, or…or what the solutions are or what the options are to adapt [TDubbs].*

## Discussion

The findings of this study showed that language discordance impacted occupational therapy service delivery in the South African public health system. National policies [20, 56] emphasise rights to language equity, including services being provided in the preferred language of the service user, because 80% of health professional-service user interactions occur across a linguistic divide [57]. This study indicates the reality is quite different to national policy, with participants using many strategies concurrently. This concurrent use offered new insights into navigating language discordance as it highlights its complexity and demonstrates that in low resource communities, one strategy is not sufficient.

Language discordance was perpetuated by systemic factors, with the overall effect that English was the main language of communication used in services. Similarly, Deumert [24] observed that English and Afrikaans are dominant languages in South African health services. This dominance valorises these languages over African languages. However, the study indicates that translanguaging occurs, which is when people use their full linguistic repertoire as a strategy in multilingual settings, ignoring rules [58]. It was used as a strategy by participants in any encounter where more than one named language was used to communicate, which is common in multilingual settings [59].

The findings of this study suggest that even with determined use of concurrent strategies, language discordance persists. Navigating it in addition to managing other challenges impacts on the quality of services, presenting practitioners with additional ethical and practical dilemmas.

### Strategies

The research aim was to describe language discordance in occupational therapy, in rural South African public health services. Participants were asked to describe the strategies they used, as language discordance is not well documented by the profession and this study was the first of its nature within occupational therapy both in South Africa and internationally*.* The findings illustrated the pragmatic and innovative approaches that the occupational therapists in a low resource context used to navigate a complex issue such as language discordance. There is evidence that other health professions use similar strategies to those identified in this study, for example, the use of interpreters and digital translation tools [8, 10, 12, 60]. In addition, the participants used: videos to demonstrate instructions; non-verbal communication including gestures and demonstrating; doing action-based therapy; collateral information from patient files; visual aids; and short phrases or words in the service user’s language. In other studies, these strategies have taken different forms such as translated information sheets [30]. Because English was widely used participants agreed that even for informal interpreters, it was important to use basic English for ease of interpretation.

Using a trained interpreter or if not available, an informal one, has been reported as the preferred and often the only strategy that health professionals used to navigate language discordance [10, 12]. However, in this study, participants did not have access to trained interpreters. The South African health system cannot afford to fund interpretation services [26], which means health professionals have to be resourceful, valuing all their strategies. While interpreters might appear to be preferable for navigating language discordance, in this study informal interpreters could not be reliably available nor was a high level of English proficiency guaranteed. By choosing fellow health professionals, concerns about ethics and professional conduct could be mitigated. Professionals were fluent in specific words about health and the services, but some English words do not have an equivalent translation in many of the South African languages. A similar observation in a Danish study indicated that translation by non-specialists presented ethical dilemmas and was blamed for misunderstandings and delays [61]. Misunderstandings could arise because of interpretation inaccuracy, especially as even trained interpreters in South Africa are generalists, lacking health-specific and profession-specific vocabulary [4, 14].

If other health professionals were not available as informal interpreters, participants minimised ethical and communication problems by using family members. Other studies indicate family members are not preferred as an interpreter [57] for various reasons, especially without prior consent from the service user. For instance, younger family members may not be informed about their parents’ or elders’ illness. Children acting as interpreters poses risks to their emotional well-being, as potentially they have to hear and interpret sensitive information [62]. However, in this study younger family members were often more proficient in English than the adults, presenting the participants with an ethical dilemma. Exploring preferred options for interpreters and other strategies with service users would be best practice, especially as there are cultural factors to consider. For example, Deumert [24] reported that male cleaners would omit or entirely change information regarding a female’s reproductive health, because it was inappropriate for a male to speak to a female about that topic or to convey bad news to a woman who is a stranger. However, informal interpreters can also act as cultural brokers between service users and health professionals, enabling decisions that are tuned into the patient, being culturally appropriate communication and reflecting their treatment preferences [63]. Thus, it makes sense to consider the wider context, taking into account factors perpetuating language discordance.

### Anglonormativity perpetuating language discordance

While health professionals have a range of strategies to address language discordance, the participants identified systemic factors perpetuating it beyond their control. Anglonormativity was drawn on to explain the dominance of English in educational trajectories where the language was positioned in an ideological and political position of power [64]. “Anglonormativity then refers to the expectation that people will be and should be proficient in English, and are deficient, even deviant, if they are not”^65:80^.

Language ideologies and linguistic elements that are often overlooked when educating students in language courses were highlighted as potentially perpetuating discordance. Participants reported a lack of sufficient preparation in schools and universities, echoing findings in studies with audiology students, speech therapy students [65], clinical psychologists [14], and community service occupational therapists [16]. These findings highlight how ideologies about language inform decisions at policy level on what languages can be taught and how language is allowed to be used [66]. African languages tend to be devalued within the education system; hence the limited time allocated within curriculum to teaching these languages. This practice continues, despite recognition that this kind of preparation is insufficient as shown above. Short-term language courses do not adequately prepare health professionals, so most participants in this study had to restart learning isiZulu, which was made easier by being immersed in the setting. Immersion is a successful way to gain proficiency in both language and culture [67] and is recommended for formal education from an early age.

Another factor perpetuating language discordance observed by participants was the effect of separating culture from language in formal education, when in practice they are so closely aligned as to be the same thing [68]. Learning more African languages would therefore also enable health professions to become more culturally competent [69], because simply knowing the literal meaning of a word does not guarantee comprehension. To graduate in South Africa, occupational therapists are required to be aware of, and sensitive to, diversity issues [70], yet the continued challenge of language discordance suggests more could be done in education.

Resistance to learning African languages, observed in this study and elsewhere, is particularly associated with white English speakers [24], perpetuating the use of English in clinical settings. Proficiency in African languages does not appear to influence choices of employment setting in South Africa, which could suggest a lack of awareness of the significance of language discordance [71]. However, most of the participants themselves were keen to improve their proficiency despite these factors, to add to their strategies for navigating language discordance.

### Navigating language discordance

Choosing the best strategies for each situation therefore required a pragmatic approach, with participants consistently using them concurrently. While a pragmatic approach is common in occupational therapy, the concurrent use of strategies is rarely reported. Al-Sharifi and colleagues [61] explored Arabic patients’ experiences in Norway, discovering the concurrent use of an interpreter and videos to convey a message. However, it was only used in two cases. The advantage of combining strategies is that communication is enhanced, benefitting service users [65]. This concurrent use of strategies provides important insights that could be drawn from by other health professionals.

Navigating language discordance was often one of trial and error. Having no guidance on how to proceed meant participants went the extra mile, solving problems by being open to learning through every interaction with service users and speakers of isiZulu. Another study of South African occupational therapy students also reported efforts to learn service users’ languages [68]. However, several participants in this study only started learning the language once they began working, probably because speaking to a service user in their home language, irrespective of their ability to communicate in English, enhanced experiences of occupational therapy as was the case for doctors in Pfaff and Couper’s study [67]. The attitude of being kind to oneself was important in the process of learning the service users’ languages, as part of trying out different strategies.

The concept of occupational resilience was used to understand the participants’ determination and flexibility. This concept was originally used in organizational psychology to describe cognitive flexibility, so that people who are occupationally resilient are adaptable and aware of their power and self-efficacy [72]. Resilience is an essential capability for educators to foster in student health professionals, to enable them to face challenges throughout their careers [73, 74] especially in the South African public health context where there are limited resources.

### Impact on occupational therapy

Despite the participants’ efforts to navigate language discordance, they believed there were negative impacts on occupational therapy. Communication is important for the profession, which depends on partnership and rapport for goalsetting and treatment to be effective. Yet the majority of the service users in South Africa receive professional health care in a language that is not their home language. In the USA, language discordance has been shown to affect communication of clinical reasoning and information sharing, as well as rapport building and goalsetting identified by participants [75].

While this study focused on occupational therapists, the negative impacts of language discordance could equally apply to health services staff more generally. The participants had to take more control, which affected service users’ access to comprehensive care. Services were less efficient, because of the additional time and effort taken to navigate language discordance. However, participants pointed out the importance of doing their best to protect the quality of services, so they were effective. It is possible that some participants being relatively inexperienced also had an impact on efficiency and effectiveness [27].

All health services aim to prevent harm, yet the participants indicated that language discordance could impact on many routine aspects of care. For example, harm could potentially be caused by less adherence to home treatment programmes, less comprehensive treatment, misunderstandings regarding appointment dates and inadvertently offending service users with inappropriate use of language [76]. In South Africa, service users have the right to access health care in a language of their choosing, so participants used informal interpreters and spoke basic and broken language. The findings suggest that these strategies were not always sufficient, and it was necessary for participants to consider other ways of preventing non-maleficence, to ensure occupational therapy offered some benefits. Their resourcefulness and determination enabled them to take a pragmatic and professional approach to language discordance, minimising as many impacts as they could.

### Limitations of the study

As the study took place at the beginning of the COVID-19 pandemic in 2020, data were collected virtually due to restrictions in place. The lack of face-to-face contact may have affected the data, although it was more convenient for the participants, being less time-consuming. The reflective journals also captured more data from the participants.

Six participants had only spent one year in the public health sector, so they had less experience of navigating language discordance in the setting. As most participants were not currently working in the setting they might not reflect in action on their strategies and the impact of language discordance. However, language discordance occurs in many healthcare settings.

## Conclusion

To navigate language discordance, the occupational therapists in this study relied on informal interpreters alongside other verbal, non-verbal and technology-assisted strategies to communicate with service users. Language discordance is complex and contextual, therefore pragmatic and flexible approaches were required, where concurrent use of strategies was the norm, indicating shortcomings of advocating for a single strategy such as providing trained interpreters. This is important for resource-allocators as resources need to be redirected to support use of multiple strategies concurrently instead of a single strategy. Minimising the impact of language discordance on the quality of services could be achieved by valuing health professionals’ resourcefulness and resilience in using their skills and characteristics. Because these are required skills and characteristics for graduation, pre-registration curricula could address navigating language discordance to apply principles of practice to a real-world setting, to better prepare new graduates for the practical and ethical challenges. Identifying language discordance as a central issue impacting on service quality might also overcome resistance to learning the service user’s language, which also enables learning about their culture. Given the rich linguistic diversity in South Africa, therapists and students could be also encouraged to draw on and value translanguaging in their concurrent strategies to navigate language discordance.

## Data Availability

The datasets generated and/or analysed during the current study are not publicly available due to the nature of the study. There is a risk that individual privacy could be compromised as the datasets include identifying content and the population from which the sample was drawn is relatively small, therefore participants could be identifiable. But are available from the corresponding author on reasonable request.
